# 变性高效液相色谱法检测多种途径获取的非小细胞肺癌组织表皮生长因子受体基因突变

**DOI:** 10.3779/j.issn.1009-3419.2010.09.03

**Published:** 2010-09-20

**Authors:** 思远 陈, 志红 陈, 爱林 郭, 健 苏, 迎 黄, 世良 陈, 绪超 张, 学宁 杨, 衿记 杨, 一龙 吴

**Affiliations:** 1 510080 广州，广东省医学科学院，广东省人民医院，广东省肺癌研究所 Guangdong Lung Cancer Institute, Guangdong General Hospital, Guangdong Academy of Medical Sciences, Guangzhou 510080, China; 2 510515 广州，南方医科大学研究生学院 Graduate School, Southern Medical University, Guangzhou 510515, China

**Keywords:** 肺肿瘤, 表皮生长因子受体, 突变, 变性高效液相色谱, Lung neoplasms, Epidermal growth factor receptor, Mutation, Denaturing high-performance liquid chromatography

## Abstract

**背景与目的:**

表皮生长因子受体(epidermal growth factor receptor, EGFR)是最重要的非小细胞肺癌(non-small cell lung cancer, NSCLC)治疗靶点，*EGFR*突变可预测酪氨酸激酶抑制剂(tyrosine kinase inhibitor, TKI)的疗效。DNA直接测序是检测*EGFR*突变最常用的方法，同时也作为突变检测的“金标准”，但该方法耗时较长、所需组织量较多且敏感性较低。变性高效液相色谱法是一种快速、自动化、高敏感性的突变检测方法，本研究旨在探讨变性高效液相色谱法(denaturing high-performance liquid chromatography, DHPLC)快速检测NSCLC肿瘤组织*EGFR*基因突变的诊断价值。

**方法:**

通过检测已知按不同比例混合的野生型及突变型EGFR质粒DNA，评价DHPLC法的准确性和敏感性。选取经多种途径获取的83例NSCLC患者的冻存肿瘤组织，提取DNA并PCR扩增EGFR外显子19、21，用DHPLC法检测并与直接测序法进行比较。

**结果:**

当突变型质粒与野生型质粒按1:100混合时，仍可被DHPLC法显著检出，而直接测序法仅可检出1:10水平。83例NSCLC组织标本中，DHPLC法检出22例*EGFR*突变(突变率26.51%)，3例直接测序法结果为野生型，余19例*EGFR*突变及61例野生型均与直接测序法结果相符。DHPLC法的敏感度为100%，特异度为95.31%，对经皮细针肺穿刺活检、淋巴结活检以及外科切除等途径获取的肿瘤样本均具有较高的敏感度、特异度。*EGFR*突变与性别、病理类型显著相关，与吸烟状态、年龄等无相关性。

**结论:**

DHPLC法可作为NSCLC患者*EGFR*基因型的初筛方法。

## Abstract (en)

**Background and objective:**

Epidermal growth factor receptor (EGFR) is the most important therapeutic target in non-small cell lung cancer (NSCLC). *EGFR* mutations may predict responsiveness to tyrosine kinase inhibitors (TKIs). These mutations are commonly identified using direct sequencing, which is considered the gold standard. But direct sequencing is time-consuming and hyposensitive. In addition, this method requires a lot of tumor specimens. Denaturing highperformance liquid chromatography (DHPLC) is a rapid automated sensitive and specific method in mutant gene detection. The aim of this study is to evaluate DHPLC as a rapid detection method for *EGFR* mutations in NSCLC tumor specimens.

**Methods:**

DHPLC was used to evaluate the accuracy and sensitivity of detection the serial dilutions of mutant and wild type EGFR plasma DNA. Frozen tumor specimens of 83 NSCLC patients from various ways had been included, after DNA extraction and polymerase chain reaction (PCR) on EGFR exon 19 and 21, the results from the direct sequencing and DHPLC were compared.

**Results:**

Mutant plasma DNA can be detected in the serial dilution of 1:100 by DHPLC and 1:10 by direct sequencing respectively. The results from DHPLC showed 22 *EGFR* mutations were detected in 83 NSCLC patients, and only 19 mutation samples had been conformed by direct sequencing. Moreover, the other 61 samples were deemed as wild type by DHPLC and direct sequencing. The sensitivity and specificity of DHPLC was 100% and 95.13% respectively. The detection of the tumor specimens from CT-guided transthoracic needle lung biopsy, lymph node biopsy and surgical resection all showed high sensitivity and specificity. *EGFR* mutation has strong correlation with gender and pathologic type, but irrelevant to age and smoking status.

**Conclusion:**

DHPLC was a precise rapid preliminary screening method for detection of NSCLC EGFR genotype.

表皮生长因子受体(epidermal growth factor receptor, EGFR)酪氨酸激酶区(tyrosine kinase, TK)已成为目前研究进展最快、最受瞩目的非小细胞肺癌(non-small cell lung cancer, NSCLC)治疗靶点，吉非替尼、厄洛替尼作为常见的酪氨酸激酶抑制剂(tyrosine kinase inhibitor, TKI)，已广泛应用于治疗NSCLC晚期患者。众多研究^[1-4]^表明，TKIs的疗效与EGFR TK区是否存在突变以及突变类型有关，据此，在对晚期NSCLC患者进行治疗决策前，明确其EGFR基因类型是非常必要的。

目前，DNA直接测序是检测*EGFR*突变最常用的方法，同时也作为突变检测的“金标准”，但该方法步骤繁琐、耗时较长、费用较高、所需组织量较多且敏感度较低，并不能满足临床需求。而变性高效液相色谱(denaturing high-performance liquid chromatography, DHPLC)是一种新的高通量筛选基因序列变异的技术，其原理是利用离子对反向高效液相色谱法在部分变性的温度条件下分离、识别野生型及突变型DNA双链，具有快速、自动化、敏感性、特异性高等特点^[5-7]^。本研究比较了DHPLC和DNA直接测序法对由CT引导经皮细针肺穿刺活检、淋巴结活检以及外科切除三种途径获取的83例NSCLC肿瘤组织EGFR基因外显子19、21突变的检测结果，进行DHPLC作为快速*EGFR*突变临床诊断平台的可行性分析，并分析了*EGFR*突变的临床意义。

## 1 对象和方法

### 1.1 研究对象及标本收集

随机选取2008年1月-2009年6月广东省人民医院收治的83例NSCLC患者的肿瘤组织，其中CT引导经皮细针肺穿刺活检标本37份、淋巴结活检标本15份以及外科切除标本31份。所有肿瘤标本离体后立即置入液氮中，后转入-80 ℃低温冰箱中保存。其中男性54例，女性29例；年龄32岁-76岁，平均年龄57.93岁；腺癌56例，鳞癌19例，其它类型8例；1999年TNM分期：Ⅰ期18例、Ⅱ期3例、Ⅲ期24例、Ⅳ期38例。所有患者获取标本前均未接受化疗或靶向治疗，相应组织经病理确诊后纳入研究。

### 1.2 DNA提取

采用上海华舜生物有限公司小量组织细胞基因组DNA快速抽提纯化试剂盒，应用组织匀浆法。经Eppendorf核酸蛋白测定仪测定DNA纯度及含量，要求光密度值OD_280_ /OD_260_＞1. 80，调整DNA浓度至10 ng/μL。

### 1.3 PCR扩增

应用PCR扩增EGFR外显子19、21的基因片段。引物使用Primer Premier 5软件自行设计，由宝生物工程(大连)有限公司合成。引物序列如下：exon19：5’-ACTGTAAAACGACGGCCAGT-3’和3’-ACCAGGAAACAGCTATGACC-5’；exon21：5’-AACACCGCAGCATGTCAAGA-3’和3’- CCTTACTTTGCCTCCTTCTGCAT-5’。反应体系：模板DNA 1 μL，上下游引物各1 μL，MarsterMix(Qiangene公司) 12.5 μL，加三蒸水至总体系共25 μL。反应条件：变性95 ℃、4 min；94 ℃、30 s，58 ℃、30 s和72 ℃、45 s，循环30次；72 ℃延伸10 min。2%琼脂糖凝胶电泳鉴定扩增片段。PCR产物分别用作直接测序法及DHPLC法检测。

### 1.4 DNA直接测序法

PCR产物按操作说明书切胶过柱纯化(QIAGEN, 28704)，纯化后的PCR产物作为模板，使用BDT V3.1测序试剂盒(Applied Biosystems)按照说明书在ABI3100测序仪(Applied Biosystems)进行测序检测。采用Chromas软件分析测序图谱，寻找*EGFR*基因外显子19、21突变区域，突变DNA阳性结果再通过反向引物测序进行验证。

### 1.5 DHPLC检测

DHPLC检测使用WAVE4500核酸片段分析系统(Transgenomic公司)，色谱柱为DNA SepCartridge分离柱，流动相为不同浓度的乙腈与三乙基铵醋酸盐(triethylamine acetate, TEAA)配制的洗脱液。PCR产物不作任何纯化处理，直接用于DHPLC分析。本研究采用了非变性条件及部分变性条件两种检测模式。其中非变性条件下WAVE4500系统运行检测温度为50 ℃，依赖DNA分子量大小分辨不同的PCR产物；部分变性条件下，PCR产物经95 ℃完全变性后逐渐冷却使其复性，若存在杂合性突变可形成异源双链，WAVE4500系统检测温度应用WAVEMAKER软件包进行预测，并根据峰型变化调整并获得最佳检测温度，外显子19、外显子21分别为60.1 ℃、63.2 ℃。分别使用本实验室构建的*EGFR*野生型、突变型质粒作阴性、阳性对照，超纯水作空白对照。DHPLC检测出现2个洗脱峰或肩峰者判别为突变型，仅出现1个洗脱峰者判别为野生型。

### 1.6 DHPLC检测的敏感性分析

#### 1.6.1 *EGFR*野生型、突变型质粒的构建

采用TA克隆法分别构建*EGFR*基因外显子19、外显子21野生型克隆和突变型(del746-750、L858R)克隆：分别提取已知EGFR外显子19突变类型为del746-750的NSCLC细胞系PC-9、EGFR外显子21突变类型为L858R的细胞系H1975以及EGFR外显子19、21均为野生型的细胞系A549的DNA为模板进行PCR，PCR产物采用凝胶回收试剂盒进行回收纯化，纯化产物与pGEM-T easy(Promega)载体连接，转化入大肠杆菌感受态细胞，37 ℃过夜培养，筛选出重组体，将重组体加至LB培养基，37 ℃摇床孵育过夜，提取质粒，测序，验证转入序列的正确性。序列正确的质粒-20 ℃冰箱中保存待用。

#### 1.6.2 DHPLC检测的敏感性分析

纯合突变型和野生型质粒的DNA浓度均调整至5 ng/μL，然后按1:1、1:5、1:10、1:20、1:50、1:100比例将二者混合，模拟临床检测中所遇到的肿瘤细胞含量不同的组织。所有不同比例的质粒DNA均分别取1 μL作为模板进行PCR扩增后直接用于DHPLC检测。外显子19分别采用非变性条件及部分变性条件进行检测，并进行比较；外显子21分别采用部分变性条件进行检测。

### 1.7 统计学分析

采用SPSS 13.0软件进行统计学分析。应用χ^2^检验比较*EGFR*突变与患者性别、吸烟状态、病理类型的关系。应用两独立样本*t*检验比较*EGFR*突变型与野生型患者之间年龄是否存在差异。以*P*＜0.05为差异有统计学意义。

## 2 结果

### 2.1 DHPLC检测的敏感性

现文献^[8-12]^报道EGFR外显子19常用非变性条件检测模式，但该模式仅能检测缺失突变，不能检测未知的点突变。本研究对EGFR外显子19按不同比例混合的野生型、del746-750缺失突变型质粒的PCR产物分别进行非变性条件及部分变性条件两种模式检测并与直接测序结果对比。当del746-750质粒、野生型质粒以1:100比例混合，突变型质粒均可被上述两种模式显著检出，而直接测序法仅可检测至1:10水平(图 1A)。EGFR外显子21只能采用部分变性条件模式检测，当L858R质粒、野生型质粒以1:100比例混合可被显著检出，而直接测序法仅可检测至1:10水平(图 1B)。

**1:**
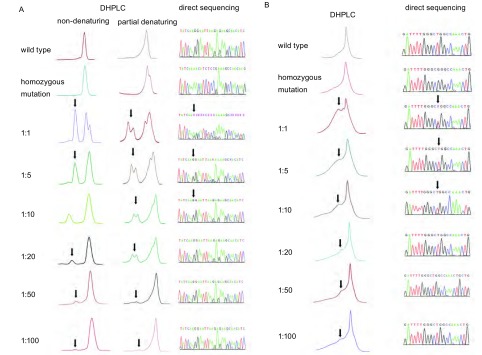
EGFR外显子19(A)和外显子21(B)检测敏感性分析，箭头提示突变峰。 Sensitivity assays for EGFR exon 19 (A) and 21 (B). The arrows indicated the mutant peak.

### 2.2 NSCLC肿瘤组织*EGFR*突变检测结果

#### 2.2.1 直接测序法检测结果

83例NSCLC肿瘤组织中检出E*GFR*突变19例，突变率为22.89%。其中外显子19缺失突变10例，共有3种基因型，delE746-A750、delL747-A749 ins P以及delL747-S752 ins S分别为6例、3例、1例。外显子21点突变9例，均为L858R。未检出其它类型突变。

#### 2.2.2 DHPLC与直接测序法对比

DHPLC法可检出22例*EGFR*突变，突变率为26.51%。其中3例直接测序法结果判读为野生型(图 2)，余19例*EGFR*突变及61例野生型均与直接测序法结果相符(图 3)，未发现DHPLC检测为野生型但直接测序为突变型的样本以及外显子19、外显子21均存在突变的样本。因为外显子19各型缺失型突变所缺失的碱基对数量相近，DHPLC检测峰型相似，难以区分不同类型的外显子19的缺失突变。由上述结果可得，DHPLC法敏感性(sensitivity)为100.00%，特异性(specificity)为95.31%，且对CT引导经皮细针肺穿刺活检、淋巴结活检以及外科切除三种常见手段所获取的肿瘤样本均具有较高的敏感度、特异度；而且阳性预测值(positive predictive value)、阴性预测值(negative predictive value)、假阳性率(false positive)、假阴性率(false negative)、诊断符合率(diagnostic accordance rate)等评价指标均达较佳水平(表 1)。

**2:**
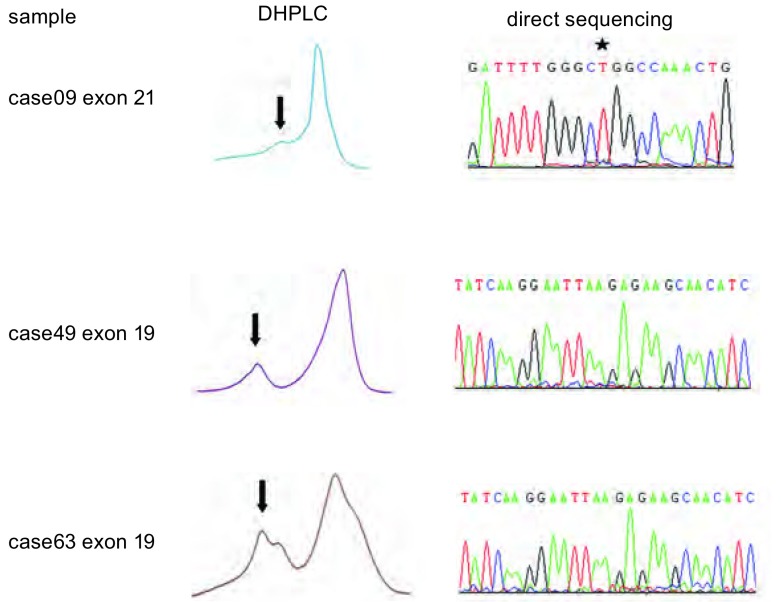
DHPLC与直接测序结果不符的检测图谱。箭头示突变峰，星号示外显子21 L858位点未见突变。 Atlas of discordant results between DHPLC and direct sequencing. The arrows indicated the mutant peaks, the asterisk indicated no mutation in exon 21 site 858.

**3:**
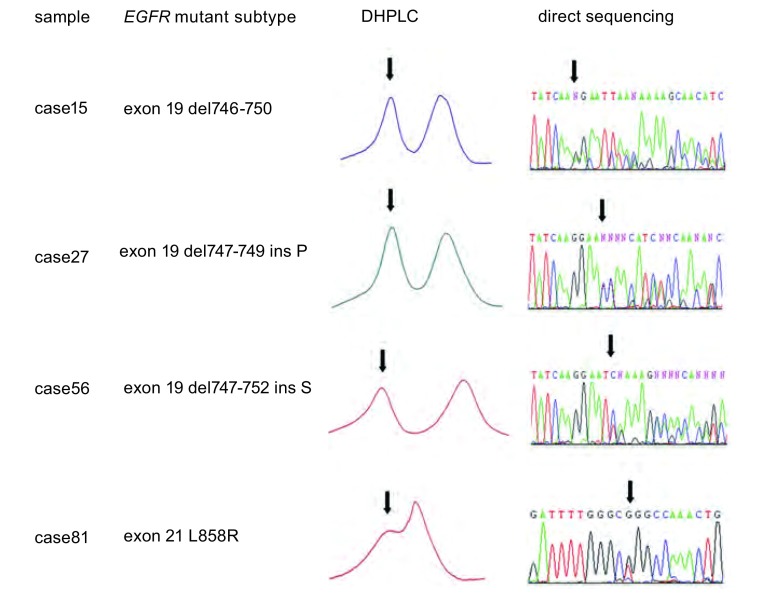
DHPLC与直接测序结果相符的检测图谱，箭头提示突变峰。 Atlas of concordant results between DHPLC and direct sequencing. The arrows indicated the mutant peaks.

**1:** DHPLC法检测NSCLC组织*EGFR*突变诊断的评价 Evaluation of DHPLC as a detection method for EGFR mutations in NSCLC specimens

Evaluation index	Sample source (%)			Total
	TNLB	TNLB	SR	
Sample size	37	15	31	83
Sensitivity	100.00	100.00	100.00	100.00
Specificity	96.55	100.00	92.00	95.31
Positive predictive value	88.89	100.00	75.00	86.36
Negative predictive value	100.00	100.00	100.00	100.00
False positive	3.45	0	8.00	4.69
False negative	0.00	0	0.00	0
Diagnostic accordance rate	97.30	100.00	93.55	96.39
TNLB: transthoracic needle lung biopsy; LNB: lymph node biopsy; SR: surgical resection.				

#### 2.2.3 *EGFR*突变与NSCLC患者临床特征的关系

本研究结果提示83例NSCLC患者*EGFR*突变率为22.89%，女性患者EGFR突变率(37.93%)高于男性患者(突变率14.81%，χ^2^=5.712，*P*=0.017)，腺癌患者*EGFR*突变率(32.14%)高于非腺癌患者(突变率3.70%，χ^2^=8.347，*P*=0.004)，差异均有统计学意义；符合女性、非吸烟、腺癌的NSCLC患者*EGFR*突变率达40.74%。但*EGFR*基因型与患者吸烟状态、年龄等无关(*P*＞0.05)。

## 3 讨论

NSCLC靶向治疗最新的进展来自以无进展生存期(progression free survival, PFS)为主要研究终点的Ⅲ期临床研究IPASS(Iressa Pan-Asia Study)，*EGFR*突变亚组吉非替尼的PFS、客观有效率(objective response rate, ORR)明显优于泰素联用卡铂(HR=0.48, 95%CI: 0.36-0.64, *P*＜0.001; 71.2% *vs* 47.3%, *P*＜0.001)，而*EGFR*野生型亚组泰素联用卡铂优于吉非替尼(HR=2.85, 95%CI: 2.05-3.98, *P*＜0.001; 23.5% *vs* 1.1%, *P*=0.001) ^[13]^，提示存在EGFR外显子19、21突变的NSCLC患者接受一线TKIs治疗，可获得较一线化疗更优的PFS和总生存时间(overall survival, OS)，且毒副作用明显较低；但对于*EGFR*野生型的患者，则很难从TKIs治疗中获益。据此，2010 NCCN NSCLC临床实践指南已推荐将厄洛替尼用于一线治疗存在*EGFR*敏感突变的NSCLC患者。自此，制定晚期NSCLC患者治疗决策前应明确*EGFR*基因是否存在突变及突变类型，存在敏感突变者给予TKIs，野生型或未知基因型者给予化疗。

目前，DNA直接测序是EGFR突变检测的“金标准”，而且是最常用的方法。但该方法步骤繁琐、耗时较长、费用较高、所需组织量较大且敏感度较低，需*EGFR*突变DNA含量占样本总DNA的20%-30%以上才可被检出。据此，有必要建立更简便、快速、价格低廉、敏感度更高且准确的*EGFR*突变检测方法。DHPLC是近年来国内外兴起的一种用于分析核苷酸片段的优秀技术平台。该系统可在同一分析标准下实现以下三种模式的运行：非变性条件：依赖分子量大小的分离；部分变性条件：根据片段大小、序列和部分变性温度的差异分离；完全变性条件：根据片段大小和序列的分离。其中部分变性条件适用于基因突变及SNP的检测，也是本研究所选取的技术平台。DHPLC具有快速、高通量、操作简单、费用低廉等优点，已广泛应用于微卫星不稳定的检测、嵌合体和低频突变的检测、SNP的检测和基因甲基化分析等。

本研究发现，DHPLC具有很高的检测敏感性，当del746-750、L858R质粒DNA仅占总DNA含量1:100时均可被显著检出，该结果与多项研究^[9, 10]^一致，证实DHPLC具较佳的重现性，可在不同的实验室进行比较。对于EGFR外显子19 DHPLC非变性条件以及部分变性条件两种检测模式均有相似的敏感性，检测峰型均易于判读。部分变性条件也适用于EGFR外显子19的检测，而且有利于发现未知突变。本研究同时应用DHPLC检测了83例NSCLC肿瘤组织EGFR外显子19、21的基因状态，与“金标准”直接测序法比较敏感度为100%、特异度95.31%、假阳性率4.69%、假阴性率为0%。而DHPLC诊断*EGFR*为野生型或突变型与金标准符合的概率即阳性预测值及阴性预测值分别为86.36%、100.00%，诊断符合率为96.39%。同时本研究中肿瘤样本分别来源于经皮肺穿刺、淋巴结活检以及外科手术切除三种临床最常用的途径，DHPLC均获得与直接测序法高度一致的结果，显示该方法对不同类型的标本均可有效地进行突变检测。DHPLC作为*EGFR*突变检测的初筛方法，与“金标准”具有高度的一致性，提示DHPLC对临床实践存在潜在的应用价值。

同时本研究结果提示83例NSCLC患者*EGFR*突变率为22.89%，女性、腺癌患者中*EGFR*突变率显著高于男性、非腺癌患者，*EGFR*突变与吸烟状态、年龄等临床特征无显著相关。

综上所述，本研究使用DHPLC系统检测了NSCLC EGFR外显子19、21突变，初步证实了该系统可应用于晚期NSCLC患者进行治疗决策前的*EGFR*基因状态初筛，并具有较高的敏感度、特异度，同时适用于多种来源的NSCLC肿瘤组织，且较直接测序法检测敏感度更高、更节约检测时间及费用，值得临床推广应用。
